# Longitudinal child data: What can be gained by linking administrative data and cohort data?

**DOI:** 10.23889/ijpds.v3i1.451

**Published:** 2018-11-14

**Authors:** Leanne C Findlay, Elizabeth Beasley, Jungwee Park, Dafna E Kohen, Yann Algan, Frank Vitaro, Richard E Tremblay

**Affiliations:** 1 Statistics Canada 150 Tunney's Pasture Driveway Ottawa, Ontario K1A 0T6; 2 CEPREMAP 48 boulevard Jourdan 75014 Paris FRANCE; 3 Sciences Po, 27, rue Saint Guillaume - 75337 Paris; 4 Groupe de recherche sur l'inadaptation psychosociale chez l'enfant (GRIP) Axe sur les maladies du cerveau Université de Montréal 3050 boul. Édouard-Montpetit, local B-234 Montréal, QC H3T 1J7

## Abstract

**Introduction:**

Linked administrative data sets are an emerging tool for studying the health and well-being of the population. Previous papers have described methods for linking Canadian data, although few have specifically focused on children, nor have they described linkage between tax outcomes and a cohort of children who are particularly at risk for poor financial outcomes.

**Objective and methods:**

This paper describes a probabilistic linkage performed by Statistics Canada linking the Montreal Longitudinal Experimental Study (MLES) and the Quebec Longitudinal Study of Kindergarten Children (QLSKC) survey cohorts and administrative tax data from 1992 through 2012.

**Results:**

The number of valid cases in the original cohort file with valid tax records was approximately 84%. Rates of false positives, false negatives, sensitivity, and specificity of the linkage were all acceptable. Using the linked file, the relationship of childhood behavioural indicators and adult income can be investigated in future studies.

**Conclusions:**

Innovative methods for creating longitudinal datasets on children will assist in examining long-term outcomes associated with early childhood risk and protective factors as well as an evidence base for interventions that promote child well-being and positive outcomes.

A true understanding of the influence of the early years on long-term individual well-being requires extensive data spanning from childhood into adulthood. Most researchers have relied on longitudinal survey designs to capture functioning in early childhood, mediating or moderating factors, and outcomes later in life (e.g., NICHD ECCD cohort [1, 2]). Surveys provide a wealth of information on a specific topic and can enlighten our understanding of complex phenomenon on specific groups of people or on the population at large. However, although longitudinal population-based surveys are rich in providing descriptive characteristics and outcome data, they can be difficult and expensive to administer. They also place a burden on respondents due to frequent contact. Furthermore, longitudinal survey data is often hampered by attrition that may impact the ability to detect associations between variables (i.e., low power) or may introduce bias if the sample is differentially lost over time [3].

On the other hand, administrative data is data collected for administrative or service-related purposes such as hospital admissions, billing, or registries. For example, vital statistics registries capture birth and death information, census data captures a count of a national population, and justice data identifies those who have been in contact with the justice system. Administrative data has the advantage of being free or low-cost, collected on the entire population (including both those at risk and those who are less likely to respond to surveys), is often updated over a long period of time, and may include “objective” reports or information that are not subject to respondent biases [3]. Moreover, administrative data often requires low respondent burden and the relatively large sample size increases the power for robust analyses. Nonetheless, since administrative data is not collected for the purpose of research, it is often lacking in basic identifiers or in-depth information to describe the individual.

To capitalize on the advantages of both survey and administrative data, an increased number of data linkages are being performed which merge individual-level survey information with population-based administrative files [e.g., 4—7]. Survey information documents the cohort in terms of descriptive characteristics, behaviour, well-being, etc.; prospective and historical administrative files can provide information on all persons in a population and thus maximize the likelihood of having complete long-term outcome data. In this way, linked files maximize the descriptive and cohort variables with long term outcomes such as educational attainment, hospitalization, participation in the justice system, or financial outcomes. The benefits of survey data can thus be married with the benefits of administrative data, creating a file that allows for a broader scope of analyses.

As an illustration, researchers using the Montreal Longitudinal Experimental Study (MLES) and the Quebec Longitudinal Study of Kindergarten Children (QLSKC) survey cohort data have linked administrative data to report educational (i.e., secondary-school degree; [8, 9]) and justice outcomes (number of criminal offenses and having a record for a violent or non-violent offense [4, 9]) for a cohort of children who participated in an early behavioural intervention program. These linked data have been used in a large number of studies to examine the relationship between childhood behaviour and experiences and young adult outcomes [for example, 10—15].

There is strong public policy interest in better understanding the mechanisms by which childhood environments and conditions may impact outcomes. For example, childhood opposition (for example, disobedience and being inconsiderate of others) in this sample has been associated in particular with theft in later years [16], and it is reasonable to expect that there may be a relationship with income. Linked data thus allow the researchers to examine long-term outcomes for the cohort that goes beyond the original longitudinal data collection.

The purpose of the current paper is to describe the methods of linking administrative tax information with child-focused survey data (in this case, the MLES and QLSKC cohorts). The advantage of this particular linked data set is that the outcomes include earnings data, by year, and over an extended time frame more so than could previously be accomplished using self-reported data. Furthermore, the linked data file allows researchers to benefit from the completeness of tax information (as opposed to tracking individuals for long-term data collection) and thus lower rates of attrition. Tax information is advantageous in that many employment earnings and income-related measures can be examined, including total income and use of social assistance programs in Canada. For the purpose of demonstrating the usefulness of the linked dataset, a final objective of the current study is to explore the association between child behaviour outcomes (from the cohort file) and later tax outcomes as an example of how this particular linked dataset can produce unique findings. 

## Methods

### Data sources

#### Tax records

The T1 Family File (T1FF) is an annual administrative database containing the income tax records of all families who file taxes in Canada. T1FF tax files were made available to Statistics Canada from the Canada Revenue Agency and preprocessed for linkage. The tax files include individual social insurance numbers which are used in the record linkage process [17] but are excluded from the analytical file. Information from the T1FF also includes a broad range of demographic and tax variables, including: year of birth, sex, marital status, number of children, year of birth of spouse, and province of residence. Income variables that were available for linkage included: wages and salaries, self-employment income, Provincial Parental Insurance Plan (PIPP) premiums, employment insurance premiums and benefits, income from social assistance, disability benefits/tax credit, total income before tax, contributions to a registered pension plan, pension adjustment, donation amount, income from workers’ compensation, income from capital gains, investment income, and union dues. Spousal income data is also included on the T1FF by using the family identification number of the respondent and the family flag of family members. All told, these variables enable researchers to define the participant’s economic outcomes including annual earnings, total personal and household income, whether or not the individual lived in low income, whether or not the individual ever claimed a charitable donation, and receipt of income assistance ever or in the past year (i.e. unemployment or social assistance).

#### MLES-QLSKC

The first study, the MLES, initially assessed the behaviour and school adjustment of 1,161 boys enrolled in kindergarten in 1983-84 and living in low income neighborhoods in Montréal, Canada [18]. The objective of the MLES was to prospectively examine the development of a sample of boys attending inner-city kindergartens in 1983-84 who had backgrounds of low socio-economic status, with a particular focus on antisocial behaviour and school adjustment. To obtain a high base rate of boys at risk for delinquent behaviour, the 53 schools with the lowest socioeconomic index were chosen and teachers were asked to rate each boy in their classes using the Social Behaviour Questionnaire [SBQ; 19]. Ratings were returned by 87% of the teachers. Questionnaires were also filled out by parents. These boys were then assessed annually to age 17, and again at ages 21, 27, and 30.

The second study, the QLSKC, is similar to the MLES but assessed 4,648 children, including both boys and girls from low, middle, and higher income neighborhoods, enrolled in kindergarten in Quebec in 1986-1987 [20]. The QLSKC was created to obtain similar data (as the MLES) on children’s behavioral outcomes from a random sample of kindergarten children attending French-speaking public schools in the province of Québec over a 2 year period (1986 and 1987). This strategy was used to obtain a sample of children that was (1) representative of all regions of Quebec, and (2) representative of urban and rural settings. A total of 4,648 students were rated on the Social Behaviour Questionnaire by both parents and teachers. A subsample of 2,000 students was assessed annually between 6 and 12 years and again at 15, 21, and 30 years. This sample provides a control group for the MLES study.

The members of these two cohorts are now in their thirties, and researchers have collected detailed longitudinal data on parent behaviours; child outcomes, behaviours, attitudes, and activities; education outcomes; psychological markers; as well as physical data. These data have been used to construct trajectories of psychological development over time. Results from both the MLES and the QLSKC have been frequently published in peer-reviewed journals.

#### Derived Record Depository (DRD)

In order to link these cohorts with the T1FF, the cohorts were first linked to the Statistics Canada’s Derived Record Depository (DRD). The DRD is a national longitudinal data base of individuals derived from a number of Statistics Canada data files and it contains only basic personal identifiers. The purpose of the DRD is to populate a Key Registry through record linkage. It is not a data source for analytical purposes, and it is an evolving data base. New individuals are routinely added and existing records are updated by linking administrative data. For the purpose of the current project, the DRD (version 4) was filtered to include only individuals born between 1977 and 1981 to match the cohort birth years. This included 2,771,101 individuals and 14,504,919There are many more records than individuals in the DRD because a person’s information (address, name, etc.) may change over time. The DRD file prepared for record linkage can contain as separate records up to 99 different combinations of identifiers for a given individual. records . The DRD contains name variables as well as parent’s surnames, birth and death dates, and geographical variables (postal code, city, CMA, CSD, province). More information about the DRD is available on the Statistics Canada Website at http://www.statcan.gc.ca/eng/sdle/status .

### Protecting respondent privacy

Statistics Canada ensures respondent privacy and confidentiality during the linkage process and subsequent use of linked files. Only employees directly involved in the linkage process have access to the unique identifying information required for linkage (such as names and birthdates) but have no access to the analysis variables. Once the data linkage process is complete, the resulting linked keys are used to create a linked file without identifying information and only the de-identified file is accessed by analysts for research purposes including validation and statistical analyses. The application for the record linkage was reviewed and approved by the Executive Management Board at Statistics Canada under the Statistics Canada Policy on Record Linkage (see http://www.statcan.gc.ca/eng/record/policy4-1 .)

### Linkage methods and results

The principal files for linkage were the combined cohort data (hereafter labeled the ‘cohort file’) from the 5804 children included in the Montreal Longitudinal Experimental Study (n=1161) and the Quebec Longitudinal Survey of Kindergarten Children (n=4643). Probabilistic record linkage was carried out using G-LinkStatistics Canada’s generalized system for record linkage 3.2 [21]. Probabilistic record linkage methodology uses non-unique identifiers (e.g., name and birth date) to calculate the likelihood that matched records refer to the same entity (e.g., an individual). This was accomplished in two steps:

#### Primary linkage

First, the cohort file was cleaned and standardized to prepare for the matching process (e.g., removing trailing blanks and accents, all characters are set to uppercase, etc.). An initial quality assessment was also performed in order to ascertain the completeness of the data (i.e., percent of data available on each matching variable) and likelihood of matching (i.e., number of distinct values). Efficiency of the record linkage process depends on relatively complete data with a relative high number of distinct values in order to discriminate between cases.

Next, a series of twenty-four conditions were created based on surnames (including parents’ surnames), given names, date of birth (and its components), province of residence, and the first three characters of postal code to limit the number of potential pairs generated. Comparison rules were created and applied to the potential pairs in order to compare surnames, given names, dates of birth and death, sex, and different geographic levels (postal code, city, census subdivision) between the two files. Based on the theory of probabilistic record linkage [22], each rule outcome was assigned a weight based on the ratio of the estimated probability of the outcome occurring for true matches to the estimated probability of the outcome occurring for non-matches. Linkage states were then assigned to the pairs based on probability ratios and thresholds. The total weight for each pair was a quantitative representation of the likelihood that the record pair was the same individual. This total weight was then compared to a lower and an upper threshold to assess whether the pair was considered definitely linked, possibly linked or not linked.

Record pairs that were determined to be definite or possible matches were then grouped to bring together all pairs that referred to the same individual. However, grouped pairs may not demonstrate the expected one-to-one mapping between the cohort and DRD; conflicts were resolved via mapping or correspondence analysis in G-Link and a linked file was created.

Overall, 98.4% (5,713 out of 5,804) of the individuals in the Quebec cohorts linked to the DRD. There were minor variations in linkage rates between the MLES and QLSKC cohorts, as well as by demographic variables (sex, birth year; see Table 1). The largest difference in linkage rates were seen when comparing cases with available geographical information (postal codes) and cases with missing geography, which was not surprising given that geography was used in the linkage process.

The T1 Personal Master File (T1PMF) of 1981 to 2012 and the Canadian Child Tax Benefits – Ident file of 2010-11 to 2013-14 were also linked to the DRD. These files provided Social Insurance Numbers (SIN) necessary to link to the T1FF.

### Secondary linkage

The cohort-DRD linkage keys and the T1PMF-DRD and CCTB-Ident-DRD linkage keys were used to link the cohort to historical tax records (all years of the T1FF between 1992 and 2012). These data provide unique information on the cohort with respect to their administratively-collected income in adulthood. For the purpose of this study, tax records from 1992 through 2012 were linked to the cohort file, although the linkage rate was low between 1992 and 1998 due to the young age of the cohort who were not likely to yet be filing taxes (i.e., less than 18 years of age). The number of valid cases in the original cohort file with valid tax records was approximately 84%. Other linkages with Canadian tax data has shown similar linkage rates between a cohort and historical tax data [23].

### Linkage accuracy

Linkage errors may create bias in the analyses, and were identified through manual review and reported as false positive rate, false negative rate, sensitivity, and specificity. False negatives occurred when matches were missed, that is, a record was not linked when it should have been. False negatives were identified from a review of all unlinked records. Fourteen of the 91 non-linked individuals were found to be good links (i.e., a false negative rate of 15.38%).

In order to measure the false positive rate, a sample size of 500 was randomly selected for different total pair weight intervals. The selected records were manually and independently reviewed by three people. The results of the manual review were reconciled and a majority-rule decision was made (at least two reviewers had to agree on the decision). False positives occurred when a record was incorrectly linked to the wrong record or when there is no true match. The false positive rate was estimated at 0.04%. The sensitivity or true positive rate was calculated as the number of pairs linked probabilistically and identified through manual review divided by the number of pairs linked through manual review. In this project, the sensitivity rate was found to be 99.76%. Specificity or true negative rate was calculated as the number of pairs not linked probabilistically nor identified through manual review divided by the number of pairs not linked through manual review. In this project, the specificity rate was 97.47%.

#### Coverage analysis

Since the Quebec cohorts were not representative of any particular population, comparisons of the linked data set to known estimates could not be made as has been done with previous linkage projects [e.g., 24]. For example, it was not possible to examine the distribution of the linked sample on certain sociodemographic characteristics (e.g., income) to the Census of Canada since the cohort was designed as an at-risk sample. However, it was possible to identify potential biases in the results by comparing the distribution of some key variables for the linked dataset, the cohorts, and the non-linked cases. Linkage rates to the DRD by demographics and variables of interest such as sex and birth year are presented in Tables 1, 2, 3. No distinct trends were noted, other than an effect of time. Fewer records were linked in 1992 through 1998 as compared to after 1998, although again this was to be expected since many individuals in the cohort would have been too young to file income taxes in those years.

### Results from analyses of the linked data

**Figure 1: Individual earnings and childhood behavior:**
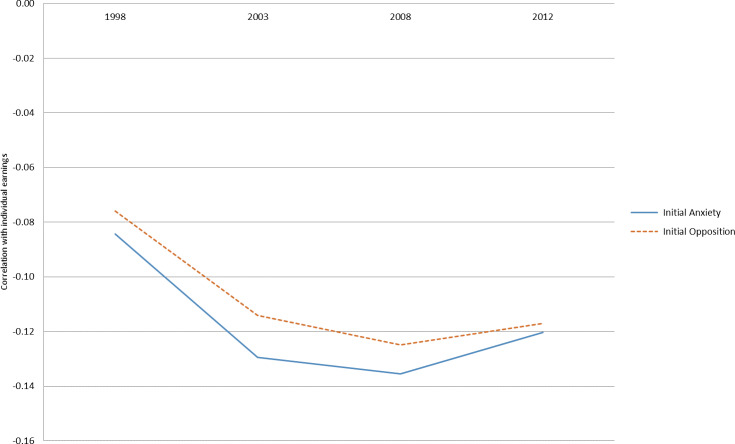


As a simple example of the value of this linked dataset, we examined the changes in the correlation of childhood behaviour measures in the cohort data and adult earnings over time from the tax data. We calculated the correlation between two childhood behaviour measures (taken in 1986 or 1987) and adult individual earnings at intervals of five years. The childhood behaviour measures were opposition and anxiety [see 19 for a description of the measures and the underlying questionnaire]. Figure 1 demonstrates the evolution of these correlations over time. Both anxiety and opposition were negatively correlated with individual earnings, and after an initial increase in negative correlation, the relationship seems to stabilize. The pattern is quite similar for anxiety and opposition behaviors in early childhood.

**Figure 2: Household income and childhood behavior:**
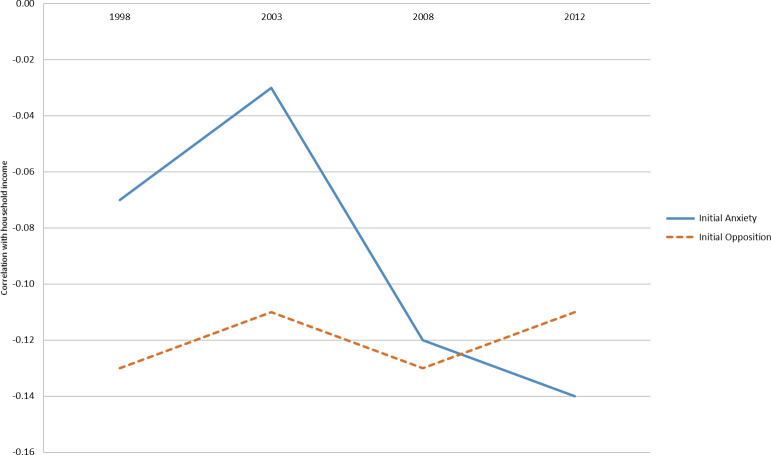


Figure 2 shows the same set of correlations for household income instead of individual earnings – that is, tax-based income of all members of the household instead of the individual as shown in Figure 1. However, the findings suggest different patterns for anxiety and opposition. In young adulthood, anxiety has a lower correlation (closer to zero) with household income than opposition, but as individuals age, the correlation is similar. In addition, the relationship of opposition to household income is fairly stable over time. These examples demonstrate the manner in which the tax income can be used for future analyses with the MLES-QLSKC cohort data in order to examine associations between early child behaviour and income-related outcomes.

## Discussion

Future research on healthy child development will increasingly rely on linked datasets to capitalize on the advantages of individual-level survey information along with prospective, population-based administrative files. In fact, Canada has several provincial linked datasets that capitalize on the linkage of several administrative data bases (e.g., Manitoba Population Research Data Repository, Population Data BC, Institute for Clinical Evaluative Sciences data in Ontario). The present study demonstrates one such linkage for child data, which links a large developmental, longitudinal cohort (which includes many child behaviour and other contextual measures) and tax information (T1FF). Using probabilistic linkage methods, Statistics Canada was able to link 84% of valid cases in the original cohort file to valid tax records. This yields a child cohort dataset with administrative tax outcomes in order to examine long term earnings and related information. This is a significant contribution to child cohort data in that previous studies have relied on self-reported income information which can be limited.

An example of the potential use of the data was provided, demonstrating the correlations between two different early child behaviours (anxiety and opposition) and individual earnings and household income. Correlations were modest, although this is roughly equivalent to the relationship between parent and child income demonstrated in several Scandinavian countries [25]. Anxiety and opposition showed similar patterns of negative correlation to individual earnings over time, but different patterns of correlation to household income: the correlation of household income with anxiety in young adulthood was lower (closer to zero) than opposition, but as individuals aged became more similar [8, 15]. One possible reason for the different patterns observed between individual earnings and household income is the possibility of belonging to a multiple earner household (through marriage or living with parents). Understanding how and why opposition and anxiety behaviours in early childhood are differently related to income over time (as well as other contextual factors included in the broader cohort data) is an interesting puzzle that can be addressed by a rich dataset such as this, which links multi-dimensional data on economic well-being (individual and household) with multi-dimensional data on childhood measures.

The correlational analysis presented above would not be possible in the absence of linked administrative data. Furthermore, self-reported income can be subject to higher levels of attrition, non-response bias, and recall errors. This linkage opens an array of analyses that were previously impossible, which can provide long-term insight into the relationship of early childhood circumstances to adult outcomes. Such studies can yield substantial new insight into the relationship of early socioeconomic status and childhood behaviour and environment to adult financial and labor market outcomes.

Despite these advantages, some limitations of linked data should be acknowledged. First, the value of linked data is dependent on complete, good quality data in the original files (in this case, cohort, DRD, and tax information) in order to optimize linkage rates between the cohort and the administrative data. Of particularly importance for the present linkage were keys on name, address, and date of birth. In this case, linkage rates were found to be quite high and of sound quality. Second, the cohort linked in this study represents a sample of children who attended kindergarten in Quebec only (and the MLES cohort only includes boys); the fact that the cohort is from Quebec facilitated linkage due to a decrease likelihood of name changes as a result of marriage. However, the results are not necessarily generalizable to the Canadian population. Further analyses may also be limited by the sample size in the cohort, and by the years of administrative data available.

### Conclusions

The linked data described in the present paper expands opportunities available with cohort data, for example, tracking childhood characteristics and early environmental and contextual variables with adult outcomes. Linked data such as this could be used to address policy-relevant questions such as the impact of socio-economic status, early behaviours, and intervention programs on adult earnings. Researchers should consider other sources of available data such as education, justice, income, health/hospitalization, and vital statistics information in developing data collection activities and plan for possible future linkages. In doing so, researchers must carefully consider respondent data, requests for sharing and linkage, and questions that can be addressed beyond the life of primary data collection activities.

**Table 1: Linkage rates for the entire Quebec cohort to the DRD:** 

	Linked records	Unlinked records	Total records	Linkage rate (%)
Cohort				
MLES	1,134	27	1,161	97.67%
QLSKC	4,579	64	4,643	98.62%
Sex				
Male	3,488	67	3,555	98.12%
Female	2,225	24	2,249	98.93%
Postal code				
Available	3,605	20	3,625	99.45%
Missing	2,108	71	2,179	96.74%
Birth year				
1977	291	5	296	98.31%
1978	843	22	865	97.46%
1979	389	4	393	98.98%
1980	1,880	29	1,909	98.48%
1981	2,310	31	2,341	98.68%
Total	5,713	91	5,804	98.43%

**Table 2: Linkage rates for the MLES cohort to the DRD:** 

	Linked records	Unlinked records	Total records	Linkage rate (%)
Intervention group missing	113	11	124	91.13%
Intent to treat	68	1	69	98.55%
Control	179	2	181	98.90%
Aggressive (no treatment)	33	0	33	100.00%
Non-disruptive	741	13	754	98.28%
Aggression-Disruptiveness Quartile 1	240	5	245	97.96%
Quartile 2	334	13	347	96.25%
Quartile 3	291	6	297	97.98%
Quartile 4	269	3	272	98.90%
Anxiety Quartile 1	246	10	256	96.09%
Quartile 2	345	8	353	97.73%
Quartile 3	268	6	274	97.81%
Quartile 4	275	3	278	98.92%
Fighting Quartile 1	530	15	545	97.25%
Quartile 2	-	-	-	-
Quartile 3	326	6	332	98.19%
Quartile 4	278	6	284	97.89%
Hyperactivity missing	4	0	4	100.00%
Quartile 1	475	17	492	96.54%
Quartile 2	154	3	157	98.09%
Quartile 3	250	5	255	98.04%
Quartile 4	251	2	253	99.21%
Inattention missing	1	0	1	100.00%
Quartile 1	277	11	288	96.18%
Quartile 2	324	8	332	97.59%
Quartile 3	286	4	290	98.62%
Quartile 4	246	4	250	98.40%
Opposition Quartile 1	359	9	368	97.55%
Quartile 2	154	6	160	96.25%
Quartile 3	379	8	387	97.93%
Quartile 4	242	4	246	98.37%
Prosocial Quartile 1	262	10	272	96.32%
Quartile 2	331	4	335	98.81%
Quartile 3	218	8	226	96.46%
Quartile 4	323	5	328	98.48%
Social withdrawal missing	5	0	5	100.00%
Quartile 1	537	14	551	97.46%
Quartile 2	-	-	-	-
Quartile 3	312	7	319	97.81%
Quartile 4	280	6	286	97.90%
Total maladjustment Quartile 1	300	11	311	96.46%
Quartile 2	269	5	274	98.18%
Quartile 3	279	9	288	96.88%
Quartile 4	286	2	288	99.31%
Birth year 1977	291	5	296	98.31%
1978	843	22	865	97.46%
Province information available	1,017	10	1,027	99.03%
Missing	117	17	134	87.31%
Postal code information available	1,017	10	1,027	99.03%
Missing	117	17	134	87.31%
Total	1,134	27	1,161	97.67%

**Table 3: Linkage rates for the QLSKC cohort to the DRD:** 

	Linked records	Unlinked records	Total records	Linkage rate (%)
Male	2,354	40	2,394	98.33%
Female	2,225	24	2,249	98.93%
Aggression missing	8	0	8	100.00%
Quartile 1	1,718	24	1,742	98.62%
Quartile 2	552	7	559	98.75%
Quartile 3	1,186	18	1,204	98.50%
Quartile 4	1,115	15	1,130	98.67%
Anxiety missing	4	0	4	100.00%
Quartile 1	1,403	18	1,421	98.73%
Quartile 2	878	10	888	98.87%
Quartile 3	1,164	17	1,181	98.56%
Quartile 4	1,130	19	1,149	98.35%
Fighting missing	5	0	5	100.00%
Quartile 1	-	-	-	-
Quartile 2	3,295	44	3,339	98.68%
Quartile 3	-	-	-	-
Quartile 4	1,279	20	1,299	98.46%
Hyperactivity missing	11	0	11	100.00%
Quartile 1	-	-	-	-
Quartile 2	2,607	34	2,641	98.71%
Quartile 3	576	6	582	98.97%
Quartile 4	1,385	24	1,409	98.30%
Inattention missing	9		9	100.00%
Quartile 1	2,001	24	2,025	98.81%
Quartile 2	564	9	573	98.43%
Quartile 3	1,006	18	1,024	98.24%
Quartile 4	999	13	1,012	98.72%
Opposition missing	10	0	10	100.00%
Quartile 1	-	-	-	-
Quartile 2	2,315	34	2,349	98.55%
Quartile 3	1,181	15	1,196	98.75%
Quartile 4	1,073	15	1,088	98.62%
Prosocial missing	11	0	11	100.00%
Quartile 1	1,118	19	1,137	98.33%
Quartile 2	1,129	15	1,144	98.69%
Quartile 3	1,395	19	1,414	98.66%
Quartile 4	926	11	937	98.83%
Birth year 1979	389	4	393	98.98%
1980	1,880	29	1,909	98.48%
1981	2,310	31	2,341	98.68%
Province information available	2,638	10	2,648	99.62%
Missing	1,941	54	1,995	97.29%
Postal code information available	2,588	10	2,598	99.62%
Missing	1,991	54	2,045	97.36%

**Table 4: Linkage rate of the Quebec cohorts to the T1FF:** 

Tax Year	MLES-DRD cohort to T1FF # of Links	MLES-DRD cohort to T1FF % of Links	QLSKC-DRD cohort to T1FF # of Links	QLSKC-DRD cohort to T1FF % of Links
1992	14	1.20%	15	1.29%
1993	60	5.16%	20	0.43%
1994	214	18.42%	53	1.14%
1995	455	39.16%	143	3.07%
1996	744	64.03%	416	8.94%
1997	941	80.98%	1262	27.12%
1998	980	84.34%	2667	57.32%
1999	972	83.65%	3837	82.46%
2000	972	83.65%	4278	91.94%
2001	979	84.25%	4328	93.02%
2002	952	81.93%	4303	92.48%
2003	964	82.96%	4311	92.65%
2004	972	83.65%	4284	92.07%
2005	967	83.22%	4281	92.01%
2006	962	82.79%	4261	91.58%
2007	962	82.79%	4289	92.18%
2008	969	83.39%	4263	91.62%
2009	946	81.41%	4269	91.75%
2010	955	82.19%	4259	91.53%
2011	962	82.79%	4264	91.64%
2012	973	83.73%	4260	91.55%

## References

[1] Vandell DL, Burchinal M, Pierce KM. Early child care and adolescent functioning at the end of high school: Results from the NICHD Study of Early Child Care and Youth Development. Dev Psychol. 2016;52(10):1634-1645 10.1037/dev0000169 27690496 PMC5115787

[2] Vaillancourt T, Miller JL, Fagbemi J, Côté S, Tremblay RE. Trajectories and predictors of indirect aggression: results from a nationally representative longitudinal study of Canadian children aged 2–10. Aggressive Behav. 2007;33(4):314-326. 10.1002/ab.20202 17593562

[3] Dahinten VS, Arim RA, Guevremont A, Kohen DE. The case for using administrative data to examine a population-based parenting intervention. Int J Child Health Hum Dev. 2014;7(2):115-124.

[4] Algan et al., in preparation

[5] Bell MF, Bayliss DM, Glauert R, Ohan JL. Using linked data to investigate developmental vulnerabilities in children of convicted parents. Dev Psychol. 2018 10.1037/dev0000521 29620388

[6] Peters PA, Tjepkema M, Finès P, Wilkins R, Crouse DL, Chan PC, Burnett RT. Data Resource Profile: 1991 Canadian Census cohort. Int J Epidemiol. 2013;42(5):1319-1326. 10.1093/ije/dyt147 24013141

[7] Sanmartin C, Decady Y, Trudeau R, Dasylva A, Tjepkema M, Finès P, et al Linking the Canadian Community Health Survey and the Canadian Mortality Database: An enhanced data source for the study of mortality. Health Rep. 2016;27(12):10-18. 28002578

[8] Vitaro F, Brendgen M, Larose S, Tremblay RE. Kindergarten disruptive behaviors, protective factors, and educational achievement by early adulthood. J Educ Psychol. 2005;97(4):617-629. 10.1037/0022-0663.97.4.617

[9] Boisjoli R, Vitaro F, Lacourse É, Barker ED, Tremblay RE. Impact and clinical significance of preventive intervention for disruptive boys: 15 year follow-up. BJPsych. 2007;191(5):415-419. 10.1192/bjp.bp.106.030007 17978321

[10] Vitaro F, Barker ED, Brendgen M, Tremblay RE. Pathways explaining the reduction of adult criminal behaviour by a randomized preventive intervention for disruptive kindergarten children. J Child Psychol Psychiatry. 2012;53(7):748-756. 10.1111/j.1469-7610.2011.02517.x 22211635

[11] Petitclerc A, Gatti U, Vitaro F, Tremblay RE. Effects of juvenile court exposure on crime in young adulthood. J Child Psychol Psychiatry. 2013;54(3):291-297. 10.1111/j.1469-7610.2012.02616.x 23009564

[12] Silva TC, Larm P, Vitaro F., Tremblay RE, Hodgins S. The association between maltreatment in childhood and criminal convictions to age 24: A prospective study of a community sample of males from disadvantaged neighbourhoods. Eur Child Adolesc Psychiatry. 2012;21(7):403-413. 10.1007/s00787-012-0281-x 22562141

[13] Véronneau M-H, Vitaro F, Pedersen S, Tremblay RE. Do peers contribute to the likelihood of secondary school graduation among disadvantaged boys? J Educ Psychol. 2008;100(2):429-442. 10.1037/0022-0663.100.2.429

[14] Hodgins S, Larm P, Ellenbogen M, Vitaro F, Tremblay RE. Teachers' ratings of childhood behaviours predict adolescent and adult crime among 3,016 males and females. Can J Psychiatry. 2013;58(3):143-150. 10.1177/070674371305800304 23461885

[15] Pingault J-B, Côté SM, Lacourse É, Galéra C, Vitaro F, Tremblay RE. Childhood hyperactivity, physical aggression and criminality: A 19-year prospective population-based study. PLoS One. 2013;8(5):1-7(e62594). 10.1371/journal.pone.0062594 PMC3641049 23658752

[16] Nagin DS, Tremblay RE. Trajectories of boys' physical aggression, opposition, and hyperactivity on the path to physically violent and nonviolent juvenile delinquency. Child Dev. 1999;70(5):1181-1196. 10.1111/1467-8624.00086 10546339

[17] Winkler WE. Record linkage Sample Surveys: Design, Methods and Applications 2009; 29A: 351-380. 10.1016/S0169-7161(08)00014-X

[18] Tremblay RE, Pagani-Kurtz L, Mâsse LC, Vitaro F, Pihl RO. A bimodal preventive intervention for disruptive kindergarten boys: Its impact through mid-adolescence. J Consult Clin Psychol. 1995;63(4):560-568. 10.1037/0022-006X.63.4.560 7673533

[19] Tremblay RE, Desmarais-Gervais L, Gagnon C, Charlebois P. The Preschool Behaviour Questionnaire: Stability of its factor structure between cultures, sexes, ages and socioeconomic classes. Int J Behav Dev. 1987;10(4):467-484. 10.1177/016502548701000406

[20] Rouquette A, Côté S, Pryor LE, Carbonneau R, Vitaro F, Tremblay RE. Cohort Profile: The Quebec Longitudinal Study of Kindergarten Children (QLSKC). Int J Epidemiol. 2014;43(1):23-33. 10.1093/ije/dys177 23159828 PMC3937968

[21] Statistics Canada. G-Link Version 3.0 User Guide (internal document).

[22] Fellegi IP, Sunter AB. A theory for record linkage. Journal of the American Statistical Association 1969;64(328):1183-1210. 10.2307/2286061

[23] Heisz A, Langevin M, Randle J. Historical data linkage of tax records on labour and income: The case of the Living in Canada Survey pilot. Longitudinal and International Study of Adults Research Paper Series, Statistics Canada 2013 Catalogue No. 89-648-X No. 002

[24] Rotermann, M. Evaluation of the coverage of linked Canadian Community Health Survey and hospital inpatient records. Health Rep. 2009; 20(1). 19388368

[25] Corak M. Income inequality, equality of opportunity, and intergenerational mobility. J Econ Perspect. 2013;27(3):79-102. 10.1257/jep.27.3.79

